# Optimization of Sparse Planar Arrays with Minimum Spacing and Geographic Constraints in Smart Ocean Applications

**DOI:** 10.3390/s19010011

**Published:** 2018-12-20

**Authors:** Shijie Hao, Feng-Xiang Ge, Xianxiang Yu, Guolong Cui, Li Ma

**Affiliations:** 1 College of Information Science and Technology, Beijing Normal University, Beijing 100875, China; hsj@mail.bnu.edu.cn; 2 School of Information and Communication Engineering, University of Electronic Science and Technology of China, Chengdu 611731, China; xianxiangy@gmail.com (X.Y.); cuiguolong@uestc.edu.cn (G.C.); 3 Key Laboratory of Acoustic Environment, Institute of Acoustics, Chinese Academy of Sciences, Beijing 100190, China; mary1968@tom.com

**Keywords:** sparse planar array, minimum spacing constraint, geographic constraint, simulated annealing (SA), robust adaptive beamforming

## Abstract

Sparse arrays can fix array aperture with a reduced number of elements to maintain resolution while reducing cost. However, grating lobe suppression, high peak side-lobe level reduction (PSLL), and constraints on the location of the array elements in the practical deployment of arrays are challenging problems. Based on simulated annealing, the element locations of a sparse planar array in smart ocean applications with minimum spacing and geographic constraints are optimized in this paper by minimizing the sum of PSLL. The robustness of the deployment-optimized spare planar array with mis-calibration is further considered. Numerical simulations show the effectiveness of the proposed solution.

## 1. Introduction

Sensor and antenna arrays play an important role in fields such as radar and sonar due to their higher processing gain and angular resolution; moreover, they are often employed in smart ocean applications for collecting information, positioning and communication. Recently, large-aperture arrays have been used widely for long-range acoustic communication in deep water [1], continuous monitoring of fish populations [2], as well as in detection, localization and classification of mechanized ocean vessels [3,4], etc. However, large-aperture arrays always require many elements with higher cost, and the deployment, impact on the ecological environment, energy supply, and maintenance of a large number of elements in the ocean are challenging problems. Therefore, sparse arrays with fewer elements are increasingly attracting interest in many applications such as target localization, ocean acoustic remote sensing [5], and passive underwater imaging [6]. In particular, sparse arrays are expected to be a potential scheme for seafloor observatory networks in oceanic engineering, radar and sonar systems with distributed arrays, etc. Furthermore, sparse arrays and optimization can also be used in the node deployment of large-scale underwater acoustic sensor networks [7,8,9] and the optimal experimental design [10,11].

A sparse array can fix array aperture with a smaller number of elements to maintain angular resolution while reducing cost. However, sparse arrays face challenges such as grating lobe suppression, high peak side-lobe level (PSLL) reduction, and even some constraints on the location of elements in the practical deployment of the array. The optimization of a sparse array is, thus, assumed to deploy its limited elements for grating lobe and peak side-lobe suppression, which is a nonlinear problem with multi-parameters, i.e., the location of every element in the sparse array. Many approaches have been proposed to solve the nonlinear optimization problem, such as particle swarm optimization (PSO) [12,13], genetic algorithms (GAs) [14,15,16], simulated annealing (SA) [17], and Bayesian compressive sampling (BCS) [18]. Certainly, some other optimization methods, such as adaptive step size random search [19], orthogonal parallel MCMC [20], and Markov chain Monte Carlo maximum likelihood [21] could also be expected to be a potential scheme for the optimization of sparse arrays. However, much research in past years has often assumed that in optimization there are no constraints on the location of array elements, which is often not the case. In practical applications, due to the minimum spacing of array elements, some optimized locations may not suitable. Therefore, a constraint on the minimum spacing between adjacent array elements needs to be imposed in the optimization [22,23]. Furthermore, in complex geographical environments on the seafloor, there are some areas in which the array elements cannot be deployed due to obstacles, e.g., ridges, trenches and boulders. Thus, a special geographic constraint must be considered for the practical applications. A method of designing sparse linear arrays (SLAs) under geographic constraints was presented in Ref. [24], but it did not consider the constraints for a sparse planar array (SPA). Generally, the size and shape of obstacles mathematically described by the geographic constraints are not fixed or even arbitrary, thus the optimization of element locations depends on the corresponding geographic constraints, and the specific adjustment for each individual obstacle is required. However, the optimization of sparse planar arrays with the minimum spacing constraints in [25,26,27,28,29] does not have the capacity to make such adaptive adjustment. In particular, sparse planar arrays are widely used for DOA estimation, have great potential for target detection and 3-D sonar imaging [30], and are more likely to face challenges due to obstacles because of their large size.

Distributed arrays are now used widely in radar and sonar systems for better performance in target detection and tracking. To achieve high accuracy in DOA estimation, many methods have been proposed, such as the ESPRIT-based algorithm [31], the 2D DOA estimation algorithm [32], and the SePDAF-based tracking method [33]. In fact, distributed arrays can be treated as sparse arrays, which will suffer cyclic ambiguity in their angle estimates, according to the spatial Nyquist sampling theorem. Thus, the optimization of sparse arrays has potential applications in the deployment of distributed array radar and sonar systems.

This paper presents the optimization of a sparse planar array in smart ocean applications with minimum spacing and geographic constraints, where the cost function is defined as the sum of PSLL for different beam shifting directions, and simulated annealing is assumed for optimization with many parameters and constraints. In particular, these two types of constraints in optimization are independently implemented in the direction of the *y*-axis and *x*-axis, respectively. Thus, the possible mutual coupling effects could be effectively avoided. Furthermore, the optimization of sparse planar arrays focuses on the deployment of array elements, which means that the location of the elements is the critical factor for a deployment-optimized sparse planar array, and the array’s performance may be sensitive to mis-calibration in practical applications. Thus, with robust adaptive beamforming used for verification, it is shown that an effective DOA estimation can be performed with a deployment-optimized sparse planar array.

## 2. Problem Formulation

### 2.1. Sparse Planar Arrays

Consider a sparse planar array with N elements in a given area of $D_{X}\times D_{Y}$. The array element locations are denoted by $\left(x_{n},y_{n}\right),\;n=0,\cdots ,N-1$. The element at $\left(x_{0},y_{0}\right)$ serves as the reference element. Without loss of generality, we let $\left(x_{0},y_{0}\right)=\left(0,0\right)$. The phase difference of arrival $\Delta \phi_{n}\left(\theta ,\varphi \right)$ between the reference element and the nth element is Ref. [34]:

(1) $$\Delta \phi_{n}\left(\theta ,\varphi \right)=\frac{2\pi }{\lambda }\left(x_{n}\sin\theta \cos\varphi +y_{n}\sin\theta \sin\varphi \right),\;n=0,\;1,\cdots ,\;N-1$$

where λ is the signal wavelength, and θ and φ are the elevation and azimuth angles, respectively. If an array in the 3-D space is considered, we have [34]:

(2) $$\Delta \phi_{n}\left(\theta ,\varphi \right)=\frac{2\pi }{\lambda }\left(x_{n}\sin\theta \cos\varphi +y_{n}\sin\theta \sin\varphi +z_{n}\cos\theta \right),$$

where $z_{n}$ denotes the nth element location on the *z*-axis.

Every Sonar or Radar in a distributed array system can be treated as a subarray of a sparse array, where all element locations of every subarray can be clustered and described by $\left(x_{n}+\Delta x_{nj},\;y_{n}+\Delta y_{nj},\;z_{n}+\Delta z_{nj}\right)$. $\Delta x_{nj}$, $\Delta y_{nj}$ and $\Delta z_{nj}$ are the distance between the reference element and the jth element of the nth subarray in the *x*-, *y*-, and *z*-axes, respectively, and are generally known. Thus, only $\left(x_{n},\;y_{n},\;z_{n}\right)$ need to be optimized in the deployment of the distributed array system.

### 2.2. Cost Function

To improve the overall performance of a sparse planar array in different beam shifting directions but with acceptable computational complexity, the sum of PSLL sampled at some scanning angles is generally taken for optimization [24,27,28,29]. The steering vector a(θ,φ) is described as:

(3) $$a\left(\theta ,\varphi \right)={\left[1,\cdots ,e^{-j\Delta \phi_{n}\left(\theta ,\varphi \right)},\cdots ,e^{-j\Delta \phi_{N-1}\left(\theta ,\varphi \right)}\right]}^{T}$$

Then:

(4) $$p_{m,i}\left(\theta ,\varphi \right)={\left|a^{H}\left(\theta ,\varphi \right)a\left(\theta_{0}\left(m\right),\varphi_{0}\left(i\right)\right)\right|}^{2},m=1,2,\cdots ,M,\;i=1,2,\cdots ,I$$

where $p_{m,i}\left(\theta ,\varphi \right)$ denotes the beampattern when the peak of the main-lobe is at $\left(\theta_{0}\left(m\right),\varphi_{0}\left(i\right)\right)$. The scanning angles $\theta_{0}$ and $\varphi_{0}$ are uniformly sampled in [−π/2,π/2] and [0,π], respectively, i.e., $\theta_{0}\left(m\right)=-\pi /2+\left(m-1\right)\cdot \pi /\left(M-1\right),\;m=1,2,\cdots ,M$, $\varphi_{0}\left(i\right)=0+\left(i-1\right)\cdot \pi /\left(I-1\right),\;i=1,2,\cdots ,I$. The sum of PSLL is defined as:

(5) $$f=\overset{I}{\underset{i=1}{\sum }}\overset{M}{\underset{m=1}{\sum }}\max\left(p_{m,i}\left(\theta ,\varphi \right)\right),$$

where the region of θ and φ for which f is valid excluding the main beam; thus, the optimization of sparse planar array can be performed by minimizing the sum of PSLL below:

(6) $$\left(\hat{x},\;\hat{y}\right)=\underset{\left(x,\;y\right)}{\min\;}\;\overset{I}{\underset{i=1}{\sum }}\overset{M}{\underset{m=1}{\sum }}\max\left(p_{m,i}\left(\theta ,\varphi \right)\right),$$

where $\left(\hat{x},\;\hat{y}\right)$ are the estimates of element locations (x, y) of the sparse planar array, $x={\left[x_{0},\;\cdots ,x_{N-1}\right]}^{T}$ and $y={\left[y_{0},\;\cdots ,y_{N-1}\right]}^{T}$.

### 2.3. Constraints

In practical applications, a sparse array is often chosen for high angular resolution with a smaller number of elements, where the minimum spacing between two elements is generally larger than λ/2. In fact, there may be no alternative in many scenarios, e.g., mutual coupling [13], element size larger than λ/2, etc. The constraint can be mathematically written as:

(7) $$\sqrt{{\left(x_{n_{1}}-x_{n_{2}}\right)}^{2}+{\left(y_{n_{1}}-y_{n_{2}}\right)}^{2}}\geq \Delta d,n_{1},n_{2}=0,\;1,\cdots ,N-1,n_{1}\neq n_{2},$$

where Δd is the minimum spacing between two elements. For convenience of optimization implementation, the minimum spacing constraint shown in Equation (7) is rewritten as:

(8) $$\max\left(\left|x_{n_{1}}-x_{n_{2}}\right|,\left|y_{n_{1}}-y_{n_{2}}\right|\right)\geq \Delta d,n_{1},n_{2}=0,\;1,\cdots ,\;N-1,n_{1}\neq n_{2},$$

which is the Chebyshev distance [27,28,29] between two elements. It is easy to prove that the minimum spacing constraint in Equation (7) is true when the requirement in Equation (8) is met, then the adjustment of the element locations in the optimization with the minimum spacing constraint can be done in only one direction (*x*- or *y*-axis), which would improve the diversity of element distribution and computational efficiency.

There may be hills, rivers, boulder, oceanic ridges and trenches, etc. in the given area, which are obstacles for array deployment; thus, geographic constraints must be considered for the optimization of a large-sized sparse planar array. In this paper, geographic obstacles are approximated in shape by their bounding rectangles, and mathematically written as:

(9) $$x_{n},y_{n}\notin \;\left(\left(G_{1_{x1}},G_{1_{x2}}\right)\cap^{\text{​}}\left(G_{1_{y1}},G_{1_{y2}}\right)\right)\cup^{\text{​}}\cdots \cup^{\text{​}}\left(\left(G_{L_{x1}},G_{L_{x2}}\right)\cap^{\text{​}}\left(G_{L_{y1}},G_{L_{y2}}\right)\right),\;n=1,\;2,\cdots N-2,\;x_{0}=0,\;y_{0}=0,x_{N-1}=D_{X},y_{N-1}=D_{Y}$$

where L is the number of obstacles in the given area of $D_{X}\times D_{Y}$. The lth obstacle is described mathematically by $\left(\left(G_{l_{x1}},G_{l_{x2}}\right)\cap^{\text{​}}\left(G_{l_{y1}},G_{l_{y2}}\right)\right)$. $\left(G_{l_{x1}},G_{l_{x2}}\right)$ and $\left(G_{l_{y1}},G_{l_{y2}}\right)$ are its ranges along the *x*- and *y*-axes, respectively, and $\cup^{\text{​}}$ and $\cap^{\text{​}}$ denote union and intersection, respectively, in set theory. The detailed mathematical description of the obstacles with other more complex shapes is shown in Ref. [35].

## 3. Constrained Optimization via Simulated Annealing

### 3.1. Implementation of Constraints in Optimization

The minimum spacing and geographic constraints in optimization would be independently implemented in the direction of the *y*-axis and *x*-axis, respectively.

Suppose the adjustment of element locations with the minimum spacing constraint given in Equation (8) is done in the direction of the *y*-axis. At the kth iteration in optimization, the element locations in the *y*-axis $y\left(k\right)={\left[y_{0}\left(k\right),\;\cdots ,y_{N-1}\left(k\right)\right]}^{T}$ are expressed as:

(10) $$y\left(k\right)=\left[\begin{array}{c} \begin{array}{c} y_{0}\left(k\right)\\ y_{1}\left(k\right) \end{array}\\ \begin{array}{c} y_{2}\left(k\right)\\ \vdots \end{array}\\ \begin{array}{c} y_{N-2}\left(k\right)\\ y_{N-1}\left(k\right) \end{array} \end{array}\right]=\left[\begin{array}{c} \begin{array}{c} {\tilde{y}}_{0}\left(k\right)\\ {\tilde{y}}_{1}\left(k\right) \end{array}\\ \begin{array}{c} {\tilde{y}}_{2}\left(k\right)\\ \vdots \end{array}\\ \begin{array}{c} {\tilde{y}}_{N-2}\left(k\right)\\ {\tilde{y}}_{N-1}\left(k\right) \end{array} \end{array}\right]+\left[\begin{array}{c} \begin{array}{c} 0\\ \Delta d \end{array}\\ \begin{array}{c} 2\Delta d\\ \vdots \end{array}\\ \begin{array}{c} \left(N-2\right)\Delta d\\ \left(N-1\right)\Delta d \end{array} \end{array}\right]=\;\tilde{y}\left(k\right)+\Delta ℰ$$

where ${\tilde{y}}_{n}\left(k\right)$ denotes the nominal location in the *y*-direction of the nth elements and $\tilde{y}\left(k\right)={\left[{\tilde{y}}_{0}\left(k\right),\cdots ,{\tilde{y}}_{N-1}\left(k\right)\right]}^{T}$,$\;\Delta ℰ={\left[0,\Delta d,\cdots ,\left(N-1\right)\Delta d\right]}^{T}$. In optimization, $y_{0}\left(k\right)\equiv 0$ and $y_{N-1}\left(k\right)\equiv D_{Y}$ are fixed, thus ${\tilde{y}}_{0}\left(k\right)\equiv 0$, ${\tilde{y}}_{N-1}\left(k\right)\equiv D_{Y}-\left(N-1\right)\Delta d$, where operator ≡ means ‘identically equal’. N−2 uniformly distributed random numbers in the interval $\left(0,D_{Y}-\left(N-1\right)\Delta d\right)$ are generated and then sorted in the ascending order as the initialized values ${\tilde{y}}_{n}\left(0\right),\;n=1,\cdots ,N-2$. Thus, $\tilde{y}\left(0\right)={\left[0,\;{\tilde{y}}_{1}\left(0\right),\;\cdots ,{\tilde{y}}_{N-2}\left(0\right),\;D_{Y}-\left(N-1\right)\Delta d\right]}^{T}$.

At the kth iteration in optimization, ${\tilde{y}}_{n}\left(k\right),\;n=1,\cdots ,N-2,$ is always updated in the interval $\left(0,D_{Y}-\left(N-1\right)\Delta d\right)$, i.e.,:

(11) $$0<{\tilde{y}}_{n}\left(k\right)<D_{Y}-\left(N-1\right)\Delta d$$

(N−1)Δd is reserved for N elements and then the minimum spacing Δd for every two adjacent element is imposed in the direction of the *y*-axis as in Equation (10). Therefore, $y_{n}\left(k\right),\;n=1,\cdots ,N-2,$ is certainly in the interval $\left(0,D_{Y}\right)$. Meanwhile:

(12) $$\left(y_{1}\left(k\right)-y_{0}\left(k\right)\right)=\left({\tilde{y}}_{1}\left(k\right)+\Delta d-y_{0}\left(k\right)\right)>\Delta d$$

(13) $$y_{N-2}\left(k\right)+\;\Delta d=\left({\tilde{y}}_{N-2}\left(k\right)+\left(N-2\right)\Delta d\right)+\Delta d<D_{Y}$$

and any two elements are separated by at least Δd.

Equations (10)–(13) make $y_{n}\left(k\right),\;n=0,\cdots ,N-1,$ could be always deployed in $\left[0,D_{Y}\right]$. But the optimization is based on ${\tilde{y}}_{n}\left(k\right)$, which ensure the minimum spacing constraint is always satisfied but the implementation of optimization is simple and flexible.

At the kth iteration in optimization with the geographic constraint, similarly, the element locations in the x-direction $x\left(k\right)={\left[x_{0}\left(k\right),\;\cdots ,x_{N-1}\left(k\right)\right]}^{T}$ are first expressed as:

(14) $$x\left(k\right)=\left[\begin{array}{c} \begin{array}{c} x_{0}\left(k\right)\\ x_{1}\left(k\right) \end{array}\\ \begin{array}{c} x_{2}\left(k\right)\\ \vdots \end{array}\\ \begin{array}{c} x_{N-2}\left(k\right)\\ x_{N-1}\left(k\right) \end{array} \end{array}\right]=\left[\begin{array}{c} \begin{array}{c} {\tilde{x}}_{0}\left(k\right)\\ {\tilde{x}}_{1}\left(k\right) \end{array}\\ \begin{array}{c} {\tilde{x}}_{2}\left(k\right)\\ \vdots \end{array}\\ \begin{array}{c} {\tilde{x}}_{N-2}\left(k\right)\\ {\tilde{x}}_{N-1}\left(k\right) \end{array} \end{array}\right]=\left[\begin{array}{c} \begin{array}{c} 0\\ {\tilde{x}}_{1}\left(k\right) \end{array}\\ \begin{array}{c} {\tilde{x}}_{2}\left(k\right)\\ \vdots \end{array}\\ \begin{array}{c} {\tilde{x}}_{N-2}\left(k\right)\\ D_{X} \end{array} \end{array}\right]=\;\tilde{x}\left(k\right)$$

where ${\tilde{x}}_{n}\left(k\right)$ denotes the nominal locations in the x-direction of the nth element and $\tilde{x}\left(k\right)={\left[{\tilde{x}}_{0}\left(k\right),\cdots ,{\tilde{x}}_{N-1}\left(k\right)\right]}^{T}$, $x_{0}\left(k\right)\equiv 0$ and $x_{N-1}\left(k\right)\equiv D_{X}$ are fixed in optimization, thus ${\tilde{x}}_{0}\left(k\right)\equiv 0$, ${\tilde{x}}_{N-1}\left(k\right)\equiv D_{X}$. The initialized values ${\tilde{x}}_{n}\left(0\right),\;n=1,\cdots ,N-2$, are random numbers uniformly distributed in the interval $\left(0,D_{X}\right)$, Thus, $\tilde{x}\left(0\right)={\left[0,{\tilde{x}}_{1}\left(0\right),\;\cdots ,{\tilde{x}}_{N-2}\left(0\right),\;D_{X}\right]}^{T}$.

At the kth iteration in optimization, the implementation of the geographic constraint is shown in Algorithm 1, where $x_{n}\left(k\right),\;n=1,\cdots ,N-2,$ is always updated in the interval $(0,D_{X}]$.

**Algorithm 1.** Implementation of Geographic Constraint1 **for**
l=1 to L
**do**2 **for**
n=1 to N−2
**do**3  **if**
$x_{n}\left(k\right)\in \left(G_{l_{x1}},G_{l_{x2}}\right)$ and $y_{n}\left(k\right)\in \left(G_{l_{y1}},G_{l_{y2}}\right)$
**then**4   $x_{n}\left(k\right)=x_{n}\left(k\right)+\left(G_{l_{x2}}-G_{l_{x1}}\right)$5   **if**
$x_{n}\left(k\right)>D_{X}$
**then**6    $x_{n}\left(k\right)=D_{X}$7   **end if**8  **end if**9 **end for**10 **end for**

The geographic constraint is independently implemented in the direction of the *x*-axis. Thus, no matter how the elements move in the direction of the *x*-axis, the implementation of the geographic constraint in optimization does not conflict with that of the minimum spacing constraint. The elements cannot be deployed in $\left(\left(G_{l_{x1}},G_{l_{x2}}\right)\cap^{\text{​}}\left(G_{l_{y1}},G_{l_{y2}}\right)\right),\;l=1,\cdots ,L$, which is the mathematical description of the lth obstacle. With the *if-then* mode in Table. 1, the nth element could be moved from the area of the lth obstacle.

### 3.2. Optimization via Simulated Annealing

Simulated annealing [17,36] is a probabilistic technique often used for approximating the global optimum of a given function depending on many parameters, e.g., array element locations in this paper. The optimization of a sparse planar array with minimum spacing and geographic constraints via simulated annealing is summarized in Algorithm 2.
**Algorithm 2.** Optimization via Simulated Annealing Initialization: $\tilde{x}\left(0\right)$, $\tilde{y}\left(0\right)$, T, K1 $x\left(0\right)=\tilde{x}\left(0\right)$, $y\left(0\right)=\tilde{y}\left(0\right)+\Delta ℰ$, compute f(0) as in Equation (5) with x(0) and y(0)2 **for**
k=0 to K−1
**do**3 **for**
n=1 to N−2
**do**4  ${\tilde{x}}_{n}^{*}\left(k\right)=\mathrm{rand}\left({\tilde{x}}_{n-1}\left(k\right),{\tilde{x}}_{n+1}\left(k\right)\right),\;{\tilde{y}}_{n}^{*}\left(k\right)=\mathrm{rand}\left({\tilde{y}}_{n-1}\left(k\right),{\tilde{y}}_{n+1}\left(k\right)\right)$5  ${\tilde{x}}^{*}\left(k\right)={\left[{\tilde{x}}_{0}\left(k\right),\cdots ,{\tilde{x}}_{n}^{*}\left(k\right),\cdots ,{\tilde{x}}_{N-1}\left(k\right)\right]}^{T},\;{\tilde{y}}^{*}\left(k\right)={\left[{\tilde{y}}_{0}\left(k\right),\cdots ,{\tilde{y}}_{n}^{*}\left(k\right),\cdots ,{\tilde{y}}_{N-1}\left(k\right)\right]}^{T}$6  $x^{*}\left(k\right)={\tilde{x}}^{*}\left(k\right)$, $y^{*}\left(k\right)={\tilde{y}}^{*}\left(k\right)+\Delta ℰ$, compute $f^{*}\left(k\right)$ as in Equation (5) with $x^{*}\left(k\right)$ and $y^{*}\left(k\right)$7 $\Delta f=f^{*}\left(k\right)-f\left(k\right)$8  **if**
Δf<0
**then**9   $f\left(k\right)=f^{*}\left(k\right)$, $\tilde{x}\left(k\right)={\tilde{x}}^{*}\left(k\right)$, $\tilde{y}\left(k\right)={\tilde{y}}^{*}\left(k\right)$, $x\left(k\right)=x^{*}\left(k\right)$, $y\left(k\right)=y^{*}\left(k\right)$10  **else**11   r=rand(0,1)12   **if**
r<exp{−Δf/T}
**then**13    $f\left(k\right)=f^{*}\left(k\right)$, $\tilde{x}\left(k\right)={\tilde{x}}^{*}\left(k\right)$, $\tilde{y}\left(k\right)={\tilde{y}}^{*}\left(k\right)$, $x\left(k\right)=x^{*}\left(k\right)$, $y\left(k\right)=y^{*}\left(k\right)$14   **end if**15  **end if**16 **end for**17 f(k+1)=f(k), $\tilde{x}\left(k+1\right)=\tilde{x}\left(k\right)$, $\tilde{y}\left(k+1\right)=\tilde{y}\left(k\right)$, x(k+1)=x(k), y(k+1)=y(k)18 T=T×0.9919 **end for**20 $\hat{x}=x\left(K\right)$, $\hat{y}=y\left(K\right)$
where rand (a,b) returns a uniformly distributed random number [36] in the interval (a, b).

## 4. Simulation Results

Assume that the SPA will be deployed in a geographic area of $D_{X}\times D_{Y}$, where $D_{X}=15$ and $D_{Y}=36$. The total number of elements N=21. The signal wavelength λ is 3 and the minimum spacing constraint Δd=λ/2. The total number of uniformly sampled elevation and azimuth scanning angles are both 7, i.e., M=I=7, the initial temperature T is 100, and the iteration number K in optimization is 1800. Three obstacles are considered, i.e., $x_{n},y_{n}\notin \left(\left(3,5\right)\cap^{\text{​}}\left(6,12\right)\right)\cup^{\text{​}}\left(\left(6,8\right)\cap^{\text{​}}\left(15,18\right)\right)\cup^{\text{​}}\left(\left(10,12\right)\cap^{\text{​}}\left(21,24\right)\right),\;n=1,\cdots ,N-2$.

**Figure 1:**
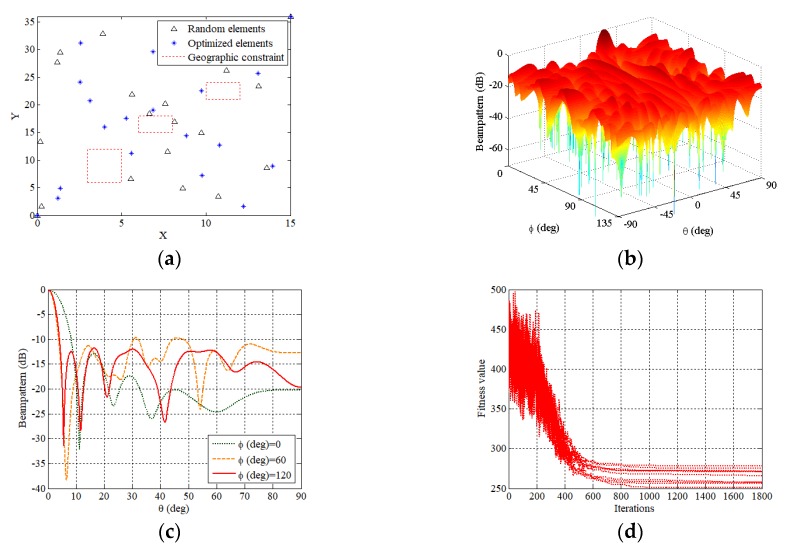
(**a**) The 21-element sparse planar arrays with random and optimized deployment that satisfy the minimum spacing and geographic constraints. (**b**) The beampattern of the optimized sparse planar array when the peak of the main-lobe is at (30°,0°). (**c**) The beampatterns in φ=0°, φ=60°, and φ=120° planes. (**d**) The convergences of the fitness value versus the number of iterations through 10 trials.

Two sparse planar arrays with random and optimized deployment are shown in Figure 1a. The elements of both sparse planar arrays are not located in areas corresponding to geographic constraints, and the minimum spacing between any two adjacent elements is greater than Δd. The beampattern of the optimized sparse planar array when the peak of the main-lobe is at (30°,0°) is given in Figure 1b. Figure 1c shows the beampatterns in φ=0°, φ=60° and φ=120° planes whose PSLLs are −12.9 dB, −9.69 dB and −11.76 dB, respectively. It is shown the optimization of sparse planar arrays with minimum spacing and geographic constraints is successfully implemented in Figure 1a–c. The good convergences of the fitness value (f in Equation (5)) versus the number of iterations through 10 trials are shown in Figure 1d.

**Figure 2:**
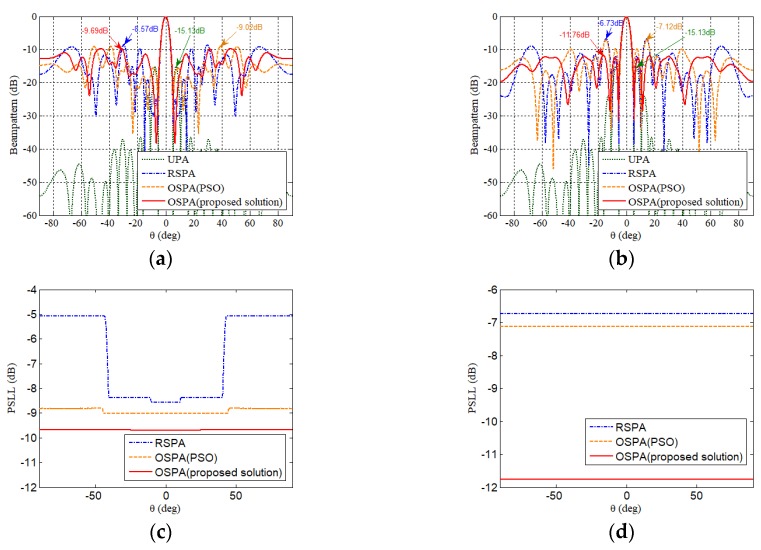
The beampatterns of the RSPA, the UPA and the OSPA by the proposed solution and PSO in (**a**) the φ=60° plane (the main-lobe is at (0°,60°) ) and (**b**) the φ=120° plane (the main-lobe is at (0°,120°) ). The PSLLs of beampatterns versus different beam shifting directions θ in the (**c**) φ=60°, and (**d**) φ=120° planes.

Figure 2a,b illustrate the beampatterns of the optimized sparse planar array (OSPA), respectively by the proposed solution and PSO [12,13,24], random sparse planar array (RSPA) without optimization, and uniform planar array (UPA) in φ=60° and φ=120° planes. The UPA has 275 elements and is also deployed in the same area. It is shown that their main-lobe beam widths are almost at the same level, which means that the spare planar array can have a larger array aperture with a smaller number of elements for high angular resolution. The PSLLs of beampatterns versus different beam shifting directions for the UPA, the RSPA, and the OSPA optimized by the proposed solution and PSO in φ=60° and φ=120° planes are illustrated in Figure 2c,d. In Figure 2, the side-lobe level of the OSPA is higher than that of the UPA but significantly lower than that of the RSPA. Generally, the low side-lobe level is very important for interference suppression in target localization.

## 5. Robustness of a Deployment-Optimized SPA

The optimization of a sparse planar array with minimum spacing and geographic constraints yields a distribution of elements optimized for high angular resolution and low side-lobe level, which means that the performance of the optimized sparse planar array in practical applications may be sensitive to mismatching, e.g., mis-calibration in hydrophone arrays or distributed underwater acoustic sensor networks, in which case the array deployment would have extremely strict accuracy requirements for the location of elements. Thus, the robustness of the deployment-optimized sparse planar array against mismatching is further considered in this paper.

Assume that the observation X(t) received by the sparse planar array in the presence of additive white Gaussian noise (AWGN) can be expressed by:

(15) X(t)=AS(t)+N(t),

where A is the steering matrix, and S(t) and N(t) correspond to the signals and noise, respectively.

The standard Capon beamformer (SCB) and robust Capon beamformer (RCB) are always taken together for performance comparison when the mismatching occurs. Generally, the RCB belongs to the class of diagonal loading techniques [37]. The SCB has the optimal weight $w_{0}={\hat{R}}_{X}^{-1}\;a_{0}/a_{0}^{H}\;{\hat{R}}_{X}^{-1}\;a_{0}$, and the weight vector of RCB is generally given by:

(16) $$w_{\mathrm{DL}}=\frac{{\left({\hat{R}}_{X}+\lambda I\right)}^{-1}a_{0}}{a_{0}^{H}{\left({\hat{R}}_{X}+\lambda I\right)}^{-1}a_{0}},$$

where ${\hat{R}}_{X}$ is the covariance matrix estimate of X(t) and ${\hat{R}}_{X}\sum_{t=1}^{N_{t}}X\left(t\right)X^{H}\left(t\right)/N_{t}$, $N_{t}$ is the number of snapshots. λ in Equation (16) is the diagonal loading value and can be often effectively determined as follows [38]:

(17) $$\mathrm{std}(\mathrm{diag}({\hat{R}}_{X}))\leq \lambda \leq \mathrm{trace}({\hat{R}}_{X})/N$$

where std(·) means the standard deviation and diag(·) means the diagonal elements of a matrix. In this paper, we let $\lambda =\mathrm{std}(\mathrm{diag}\left({\hat{R}}_{X}\right))$.

**Figure 3:**
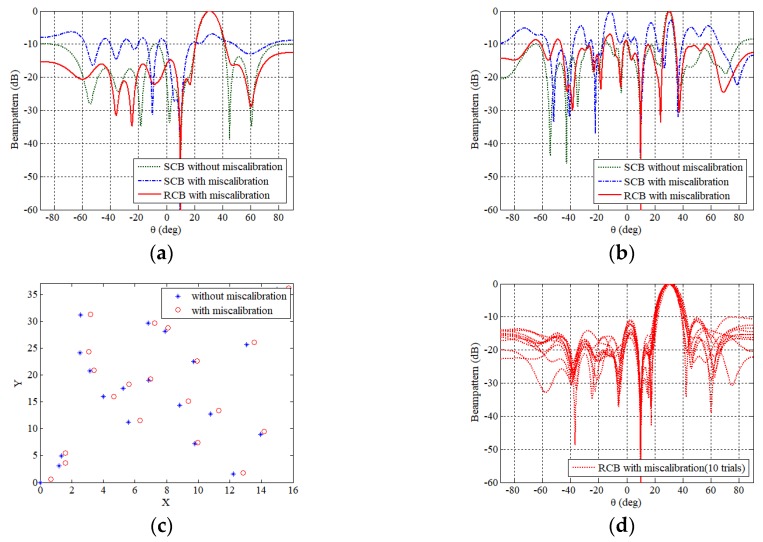
Comparison of the beampatterns in the (**a**) φ=0° and (**b**) φ=60° plane of the SCB without mis-calibration, the SCB and the RCB with mis-calibration. (**c**) The elements’ locations with and without mis-calibration. (**d**) The beampatterns of RCB with mis-calibration through 10 trials.

The performance comparison is given in Figure 3a,b, where standard Capon beamforming (SCB) without mis-calibration, SCB with mis-calibration, and robust Capon beamforming (RCB) with mis-calibration are presented. The mis-calibration in simulations is assumed to have a Gaussian distribution with a standard deviation of Δ*d*/2. The elements are illustrated in Figure 3c. It is shown the OSPA with miscalibration still has good performance by taking the robust adaptive beamforming. Figure 3d shows the beampatterns of RCB with mis-calibration through 10 trials.

## 6. Conclusions

In this paper, the optimization of SPAs in smart ocean applications with minimum spacing and geographic constraints has been presented, where the cost function is defined as the sum of PSLL for different beam shifting directions and simulated annealing has been used for the optimization. The implementation of these constraints in optimization is also given. Furthermore, the robustness of the deployment-optimized SPA against mis-calibration of the array in practical applications is also verified. Simulations show that the optimized SPA is effective for the DOA estimation.
